# Supplementary material for: Heterogeneity in strategy use during arbitration between experiential and observational learning
Source: Nat Commun. 2024 May 24;15:4436. doi: 10.1038/s41467-024-48548-y (PMC11126711; doi:10.1038/s41467-024-48548-y)
Supplement: Supplementary file 1 — Supplementary Information [file 41467_2024_48548_MOESM1_ESM.pdf]

Supplementary Information

Heterogeneity in strategy use during arbitration between experiential and observational learning

Caroline J. Charpentier[1,2]*, Qianying Wu[1], Seokyoung Min[1], Weilun Ding[1], Jeffrey Cockburn[1], John P. O'Doherty[1]

[1]Division of Humanities and Social Sciences, California Institute of Technology, Pasadena, CA, USA

[2]Department of Psychology & Brain and Behavior Institute, University of Maryland, College Park, MD, USA

*Correspondence to: ccharpen@caltech.edu

A) ME-GLM to assess hybrid behavior **(Fig. 2E-F)**

$$choice \sim 1 + out + pa + (1 + out + pa \mid subID)$$

	Study 1			Study 2		
Predictor	*Estimate ±SE*	*t(19862)*	*95% CI*	*Estimate ±SE*	*t(77960)*	*95% CI*
**Intercept**	-0.044±0.027	-1.65	[-0.10, 0.01]	-0.066±0.011	-6.06	[-0.09, -0.05]
**Past outcome (EL)**	0.357 ±0.051	6.94	[0.26, 0.46]	0.519 ±0.025	20.74	[0.47, 0.57]
**Past action (OL)**	0.216 ±0.026	8.21	[0.16, 0.27]	0.241 ±0.012	19.60	[0.22, 0.27]

B) ME-GLM to assess interaction with OL uncertainty **(Fig. 3C-D left)**

$$choice \sim 1 + out_{low}^{OLu} + out_{high}^{OLu} + pa_{low}^{OLu} + pa_{high}^{OLu} + (1 + out_{low}^{OLu} + out_{high}^{OLu} + pa_{low}^{OLu} + pa_{high}^{OLu} \mid subID)$$

	Study 1			Study 2		
Predictor	*Estimate ±SE*	*t(19860)*	*95% CI*	*Estimate ±SE*	*t(77958)*	*95% CI*
**Intercept**	-0.048±0.028	-1.70	[-0.10, 0.01]	-0.068±0.012	-5.86	[-0.09, -0.05]
**Past outcome – low OL unc**	0.516 ±0.061	8.45	[0.40, 0.64]	0.712 ±0.03	24.10	[0.65, 0.77]
**Past outcome – high OL unc**	0.226 ±0.056	4.05	[0.12, 0.33]	0.396 ±0.029	13.56	[0.34, 0.45]
**Past action – low OL unc**	0.609 ±0.068	8.93	[0.48, 0.74]	0.664 ±0.029	22.70	[0.61, 0.72]
**Past action – high OL unc**	-0.136±0.036	-3.80	[-0.21, -0.07]	-0.14 ±0.019	-7.53	[-0.18, -0.10]

C) ME-GLM to assess interaction with EL uncertainty **(Fig. 3C-D right)**

$$choice \sim 1 + out_{low}^{OLu} + out_{high}^{OLu} + pa_{low}^{OLu} + pa_{high}^{OLu} + (1 + out_{low}^{OLu} + out_{high}^{OLu} + pa_{low}^{OLu} + pa_{high}^{OLu} \mid subID)$$

	Study 1			Study 2		
Predictor	*Estimate ±SE*	*t(19860)*	*95% CI*	*Estimate ±SE*	*t(77958)*	*95% CI*
**Intercept**	-0.044±0.027	-1.64	[-0.10, 0.01]	-0.067±0.011	-6.11	[-0.09, -0.05]
**Past outcome – low EL unc**	0.521 ±0.068	7.67	[0.39, 0.65]	0.732 ±0.034	21.22	[0.66, 0.80]
**Past outcome – high EL unc**	0.203 ±0.041	4.93	[0.12, 0.28]	0.324 ±0.020	16.58	[0.29, 0.36]
**Past action – low EL unc**	0.121 ±0.027	4.56	[0.07, 0.17]	0.087 ±0.013	6.82	[0.06, 0.11]
**Past action – high EL unc**	0.289 ±0.037	7.86	[0.22, 0.36]	0.364 ±0.018	20.60	[0.33, 0.40]

D) ME-GLM to assess interaction with magnitude **(Fig. S2C-D)**

$$choice \sim 1 + out_{low}^{OLu} + out_{high}^{OLu} + pa_{low}^{OLu} + pa_{high}^{OLu} + (1 + out_{low}^{OLu} + out_{high}^{OLu} + pa_{low}^{OLu} + pa_{high}^{OLu} \mid subID)$$

	Study 1			Study 2		
Predictor	*Estimate ±SE*	*t(19860)*	*95% CI*	*Estimate ±SE*	*t(77958)*	*95% CI*
**Intercept**	-0.042±0.027	-1.58	[-0.09, 0.01]	-0.063±0.011	-5.94	[-0.08, -0.07]
**Past outcome – high mag**	0.515 ±0.074	6.91	[0.37, 0.66]	0.748 ±0.036	20.93	[0.68, 0.82]
**Past outcome – low mag**	0.092 ±0.025	3.74	[0.04, 0.14]	0.166 ±0.014	11.54	[0.14, 0.19]
**Past action – high mag**	0.199 ±0.032	6.28	[0.14, 0.26]	0.216 ±0.015	14.85	[0.19, 0.24]
**Past action – low mag**	0.222 ±0.030	7.34	[0.16, 0.28]	0.244 ±0.015	16.56	[0.22, 0.27]

Table S1. Summary of ME-GLM results and statistics.

A) $\boldsymbol{OL\ choice \sim 1 + OLunc_{trial} + OLunc_{condition} + (1 + OLunc_{trial} + OLunc_{condition} \mid subID)}$

	Study 1			Study 2		
Predictor	*Estimate ±SE*	*t(19991)*	*95% CI*	*Estimate ±SE*	*t(77960)*	*95% CI*
**Intercept**	-0.116±0.042	-2.77	[-0.20, -0.03]	-0.093±0.021	-4.50	[-0.13, -0.05]
**OL unc trial**	0.726 ±0.090	8.04	[0.55, 0.90]	0.780 ±0.041	19.07	[0.70, 0.86]
**OL unc condition**	0.109 ±0.035	3.11	[0.04, 0.18]	0.150 ±0.019	8.12	[0.11, 0.19]

B) $\boldsymbol{EL\ choice \sim 1 + ELunc_{trial} + ELunc_{condition} + (1 + ELunc_{trial} + ELunc_{condition} \mid subID)}$

	Study 1			Study 2		
Predictor	*Estimate ±SE*	*t(19991)*	*95% CI*	*Estimate ±SE*	*t(77960)*	*95% CI*
**Intercept**	0.002 ±0.026	0.08	[-0.05, 0.05]	0.094 ±0.014	6.53	[0.07, 0.12]
**EL unc trial**	0.391 ±0.045	8.42	[0.30, 0.48]	0.465 ±0.025	18.41	[0.42, 0.51]
**EL unc condition**	0.025 ±0.033	0.76	[-0.04, 0.09]	0.058 ±0.016	3.61	[0.03, 0.09]

Table S2. Summary of ME-GLMs predicting choice from uncertainty trials and uncertainty conditions.

Model	***Description***	$\boldsymbol{N_{param}}$	***AIC***	***OOS accuracy***
**ExpLearn_nomag**	Magnitude is ignored, only learning of reward probability and outcomes are treated as binary (1: reward, 0: no reward)	2	212.3	0.524
**ExpLearn_mag**	Magnitude is incorporated in EV calculation and learned.	2	210.7	0.525
**ExpLearn_decay**	Magnitude is learned separately and boosts EV, decay rate (free parameter) applied to unchosen and unrewarded tokens.	4	208.7	0.539
**ExpLearn**	Model variant used in the main analyses reported in the manuscript. It is the same as ExpLearn_decay but with a fixed decay rate of 0.5.	3	**207.7**	**0.539**

Table S3. Summary of additional EL models tested during initial exploratory analyses of Study 1 data. We found that the ExpLearn model performed best when accounting for model complexity, which is why we selected this model as our candidate model for the EL mechanism. We also found that there was no statistically significant difference between the mean decay rate estimated from the ExpLearn_decay model and 0.5 (mean=0.513, sd=0.227, T(125)=0.639, P=0.52), hence, providing a rationale for why we decided to use a fixed decay of 0.5 in our final EL model, thus minimizing model complexity.

***A) Learning curve per group* (Fig. 5D-E)**

accuracy ~ trial*group + gender + age + education + ICAR_score + (1|subID)

	Study 1			Study 2		
Predictor	*F*	*DF (num, den)*	*P-value*	*F*	*DF (num, den)*	*P-value*
**trial**	34.06	(1, 875)	<0.001	62.09	(1, 3423)	<0.001
**group**	5.30	(4, 676.26)	<0.001	20.39	(4, 2420.2)	<0.001
**gender**	1.28	(2, 125)	0.28	0.39	(2, 489)	0.680
**age**	0.36	(1, 125)	0.55	0.001	(1, 489)	0.972
**education**	0.014	(1, 125)	0.90	0.06	(1, 489)	0.801
**ICAR score**	0.14	(1, 125)	0.71	0.22	(1, 489)	0.640
**trial*group**	13.49	(4, 875)	<0.001	18.81	(4, 3423)	<0.001

***B) OL and EL main effects per group* (Fig. 6A-B)**

glme_effect ~ strategy*group + gender + age + education + ICAR_score + (1|subID)

	Study 1			Study 2		
Predictor	*F*	*DF (num, den)*	*P-value*	*F*	*DF (num, den)*	*P-value*
**strategy**	41.07	(1, 125)	<0.001	216.8	(1, 489)	<0.001
**group**	40.74	(4, 125)	<0.001	106.4	(4, 489)	<0.001
**gender**	1.07	(2, 125)	0.35	0.63	(2, 489)	0.531
**age**	0.09	(1, 125)	0.76	0.04	(1, 489)	0.846
**education**	0.006	(1, 125)	0.94	1.21	(1, 489)	0.272
**ICAR score**	0.008	(1, 125)	0.93	6.45	(1, 489)	0.011
**strategy*group**	25.55	(4, 125)	<0.001	73.73	(4, 489)	<0.001

***C) Interaction with OL uncertainty per group* (Fig. 7C,E)**

glme_effect ~ strategy*OLunc*group + gender + age + education + ICAR_score + (1|subID)

	Study 1			Study 2		
Predictor	*F*	*DF (num, den)*	*P-value*	*F*	*DF (num, den)*	*P-value*
**strategy**	36.48	(1, 375)	<0.001	212.43	(1, 1467)	<0.001
**OLunc**	219.42	(1, 375)	<0.001	620.53	(1, 1467)	<0.001
**group**	37.13	(4, 125)	<0.001	109.97	(4, 489)	<0.001
**gender**	1.07	(2, 125)	0.35	0.77	(2, 489)	0.462
**age**	0.31	(1,125)	0.58	0.45	(1, 489)	0.504
**education**	0.004	(1, 125)	0.95	0.73	(1, 489)	0.393
**ICAR score**	0.09	(1, 125)	0.76	4.05	(1, 489)	0.045
**strategy*OLunc**	29.38	(1, 375)	<0.001	81.09	(1, 1467)	<0.001
**strategy*group**	29.19	(4, 375)	<0.001	73.90	(4, 1467)	<0.001
**OLunc*group**	32.08	(4, 375)	<0.001	76.93	(4, 1467)	<0.001
**strategy*OLunc*group**	20.22	(4, 375)	<0.001	33.50	(4, 1467)	<0.001

***D) Interaction with EL uncertainty per group* (Fig. 7D,F)**

glme_effect ~ strategy*ELunc*group + gender + age + education + ICAR_score + (1|subID)

	Study 1			Study 2		
Predictor	*F*	*DF (num, den)*	*P-value*	*F*	*DF (num, den)*	*P-value*
**strategy**	102.05	(1, 375)	<0.001	516.56	(1, 1467)	<0.001
**ELunc**	23.85	(1, 375)	<0.001	32.37	(1, 1467)	<0.001
**group**	40.28	(4, 125)	<0.001	113.40	(4, 489)	<0.001
**gender**	0.79	(2, 125)	0.45	0.58	(2, 489)	0.560

**age**	0.04	(1, 125)	0.83	0.09	(1, 489)	0.763
**education**	0.01	(1, 125)	0.91	0.66	(1, 489)	0.418
**ICAR score**	0.003	(1, 125)	0.96	6.79	(1, 489)	0.009
**strategy*ELunc**	171.32	(1, 375)	<0.001	666.37	(1, 1467)	<0.001
**strategy*group**	53.90	(4, 375)	<0.001	155.64	(4, 1467)	<0.001
**ELunc*group**	6.13	(4, 375)	<0.001	17.42	(4, 1467)	<0.001
**strategy*ELunc*group**	19.90	(4, 375)	<0.001	97.38	(4, 1467)	<0.001

E) Interaction with magnitude per group **(Fig. S2E,F)**

glme_effect ~ strategy*magnitude*group + gender + age + education + ICAR_score + (1|subID)

	**Study 1**			**Study 2**		
Predictor	*F*	*DF (num, den)*	*P-value*	*F*	*DF (num, den)*	*P-value*
**strategy**	35.33	(1, 375)	<0.001	266.68	(1, 1467)	<0.001
**magnitude**	108.49	(1, 375)	<0.001	414.36	(1, 1467)	<0.001
**group**	40.40	(4, 125)	<0.001	108.94	(4, 489)	<0.001
**gender**	1.08	(2, 125)	0.34	0.58	(2, 489)	0.561
**age**	0.13	(1, 125)	0.72	0.004	(1, 489)	0.949
**education**	0.003	(1, 125)	0.96	1.39	(1, 489)	0.239
**ICAR score**	0.06	(1, 125)	0.81	6.20	(1, 489)	0.013
**strategy*magnitude**	127.14	(1, 375)	<0.001	465.93	(1, 1467)	<0.001
**strategy*group**	28.30	(4, 375)	<0.001	109.09	(4, 1467)	<0.001
**magnitude*group**	31.10	(4, 375)	<0.001	64.47	(4, 1467)	<0.001
**strategy*mag.*group**	36.58	(4, 375)	<0.001	99.86	(4, 1467)	<0.001

Table S4. Summary of between-subjects mixed linear model effects assessing group differences. Five separate models were run using *lmer* in R, followed by Type III analysis of variance (F-test) with Satterthwaite's method to assess significance. No adjustment for multiple comparison were made across models (given that the dependent variable is different between models) or across predictors (given that all predictors are already competing for variance in each model).

A) Factor scores per group **(Fig. 8A-E)**

score ~ factor*group + gender + age + education + ICAR_score + study + (1|subID)

Predictor	*F*	*DF (num, den)*	*P-value*
**factor**	0.57	(7, 3948)	0.779
**group**	0.23	(4, 564)	0.920
**gender**	0.71	(2, 564)	0.493
**age**	25.40	(1, 564)	<0.001
**education**	22.41	(1, 564)	<0.001
**ICAR score**	1.38	(1, 564)	0.240
**study**	0.02	(1, 564)	0.888
**factor*group**	2.38	(28, 3948)	<0.001

B) Questionnaire summary scores per group **(control analysis)**

score ~ questionnaire*group + gender + age + education + ICAR_score + study + (1|subID)

Predictor	*F*	*DF (num, den)*	*P-value*
**questionnaire**	0.52	(4, 2256)	0.723
**group**	0.19	(4, 564)	0.944
**gender**	1.32	(2, 564)	0.269
**age**	19.79	(1, 564)	<0.001
**education**	26.95	(1, 564)	<0.001
**ICAR score**	1.44	(1, 564)	0.231
**study**	0.0008	(1, 564)	0.977
**questionnaire*group**	1.40	(16, 2256)	0.131

Table S5. Summary of between-subjects mixed linear model effects assessing group differences in psychiatric symptom dimensions. Following pooling between the two datasets, a mixed linear model was run to determine whether factor scores differ across groups, as shown by the significant factor*group interaction in **A**, controlling for gender, age, education, ICAR score and study. Running the same analysis with questionnaire summary scores instead of factors (**B**), shows no significant interaction, thus further justifying the factor analysis approach to better separate meaningful symptom dimensions. The two regression models were run using *lmer* in R, followed by Type III analysis of variance (F-test) with Satterthwaite's method to assess significance. No adjustment for multiple comparison were made across models (given that the dependent variable is different between models) or across predictors (given that all predictors are already competing for variance in each model).

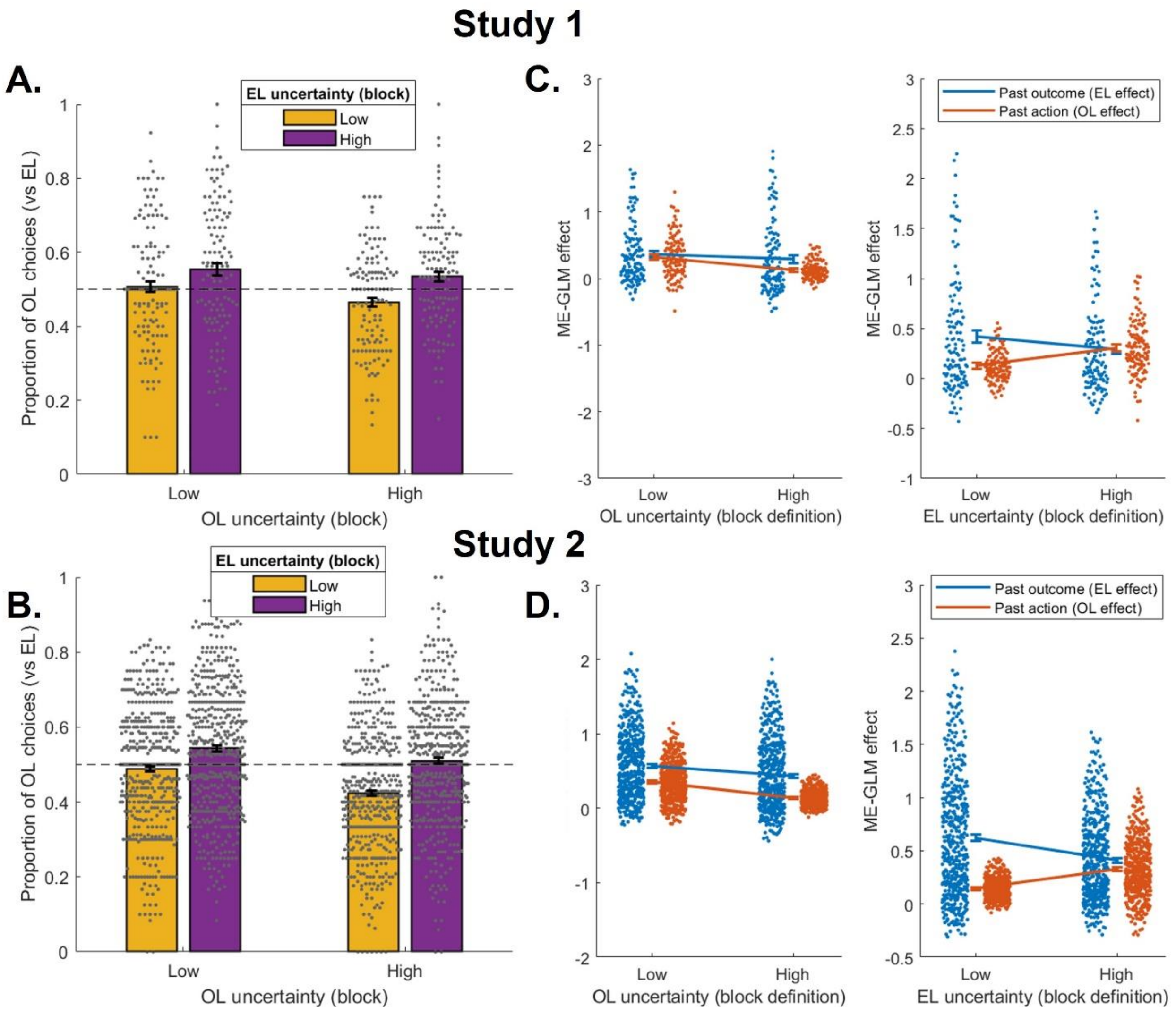

Figure S1. Behavioral signature of uncertainty-driven arbitration between EL and OL. This figure is meant to be compared with main text **Figure 3**, and shows the same analysis except that it uses the condition (or block) definition of OL and EL uncertainty – that is, the manipulation of contingencies established in the task design – instead of the trial-by-trial definition of uncertainty, meant to be more representative of changes of uncertainty as directly experienced by the participants. As a sanity check, this analysis shows that the OL and EL uncertainty conditions do influence choice in the expected direction. However, the effects are weaker than when examining trial-by-trial uncertainty variations (see **Figure 3** for comparison). Study 1 (**A, C**): N=126 independent participants; Study 2 (**B, D**): N=493 independent participants.

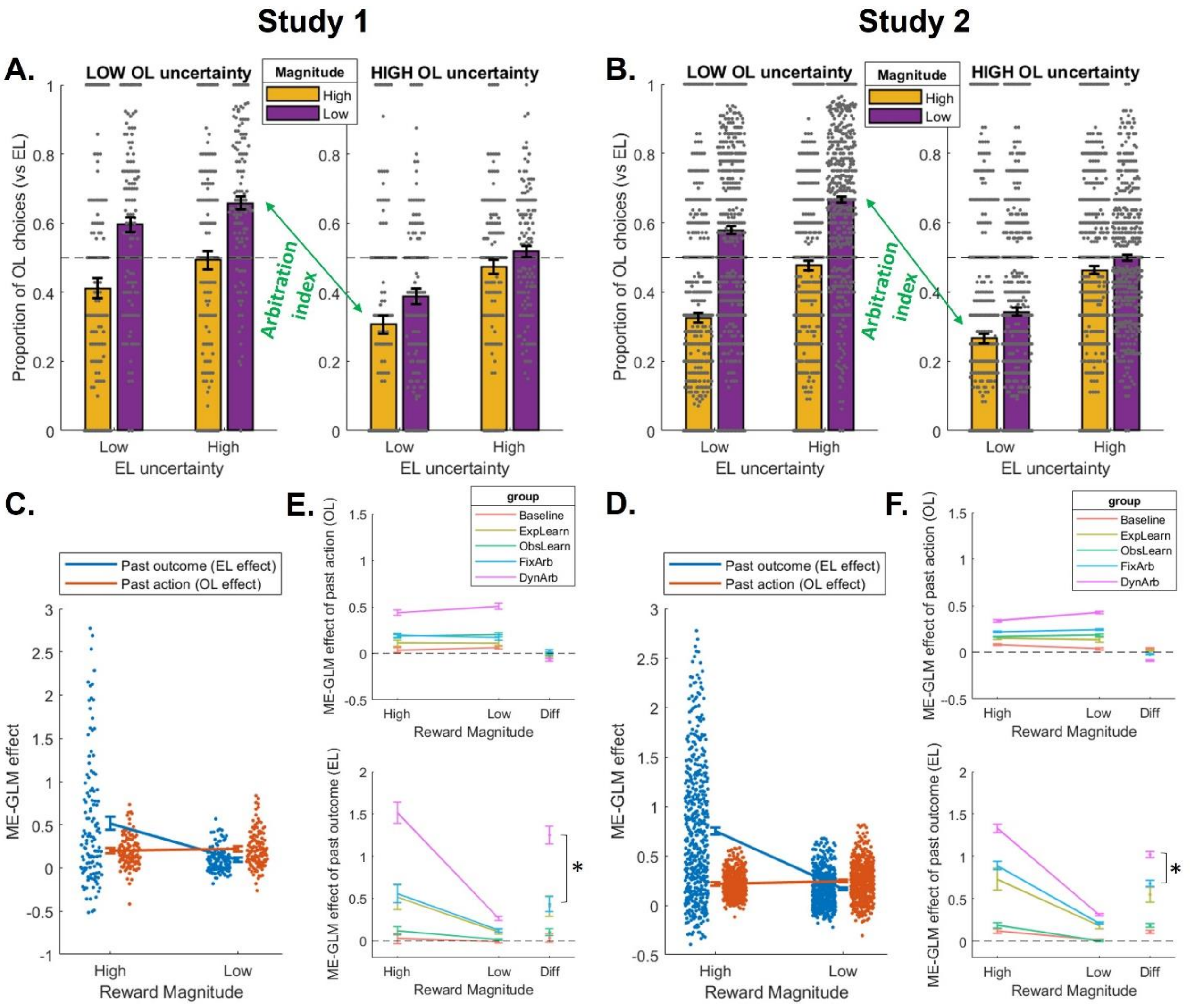

Figure S2. Effects of reward magnitude on arbitration. A-B. The proportion of OL choices was computed like in **Fig. 3A-B**, but further broken down by past reward magnitude (low or high), for Study 1 (**A**, N=126) and Study 2 (**B**, N=493), thus leading to a 2 (OL uncertainty: low/high) by 2 (EL magnitude: low/high) by 2 (magnitude: low/high) analysis. In addition to the effects reported in the main text, we also found a main effect of reward magnitude on OL choice propensity (Study 1: $F(1,833)=58.52$, $P<0.001$, $\eta_p^2=0.066$; Study 2: $F(1,3234)=269.89$, $P<0.001$, $\eta_p^2=0.077$). Each dot is an individual participant; error bars represent SEM. The difference between trials where OL is maximal (low OL uncertainty, high EL uncertainty, low magnitude) and trials where EL is maximal (high OL uncertainty, low EL uncertainty, high magnitude), depicted by the green arrow, was calculated for each individual as an index of arbitration (reported in **Fig. 7A-B**). **C-D.** A mixed-effects GLM was run to quantify the effect of past high and low reward magnitude on EL (blue) and OL (orange) separately (see **Table S1D** for details and statistics), for Study 1 (**C**, N=126) and Study 2 (**D**, N=493). Data represent the fixed effect coefficient estimates between high and low magnitude; error bars represent the standard error associated with those estimates; and each dot is an individual participant (random effect). A significant strategy*magnitude interaction was found (Study 1: $F(1,375)=62.79$,$P<0.001$, $\eta_p^2=0.143$; Study 2: $F(1,1467)=535.32$, $P<0.001$, $\eta_p^2=0.267$), such that high reward magnitude increased the tendency to rely on EL but had no effect on the tendency to rely on OL. **E-F.** The random effects obtained from **C-D** were averaged separately for each group, for Study 1 (**E**, $N_{Baseline}=25$, $N_{ExpLearn}=21$, $N_{ObsLearn}=40$, $N_{FixArb}=14$, $N_{DynArb}=26$) and Study 2 (**F**, $N_{Baseline}=83$, $N_{ExpLearn}=24$, $N_{ObsLearn}=95$, $N_{FixArb}=160$, $N_{DynArb}=131$), thus showing the effect of reward magnitude on the GLM effects of past partner action (OL effect; **top**) and past outcome (EL effect; **bottom**). See **Table S4E** for statistics. The difference between high and low magnitude is also

depicted, allowing for a direct comparison of the arbitration signature between groups. Error bars represent SEM for each group. When the dynamic arbitration group showed the most extreme signature of arbitration, a Welch two-sample two-sided t-test was run to specifically test that it was stronger than in the fixed mixture group; stars depict significant differences (Study 1: $t(29.061)=5.38$, 95% CI [0.059, 0.132], $P<0.001$; Study 2: $t(240.39)=9.52$, 95% CI [0.053, 0.081], $P<0.001$).

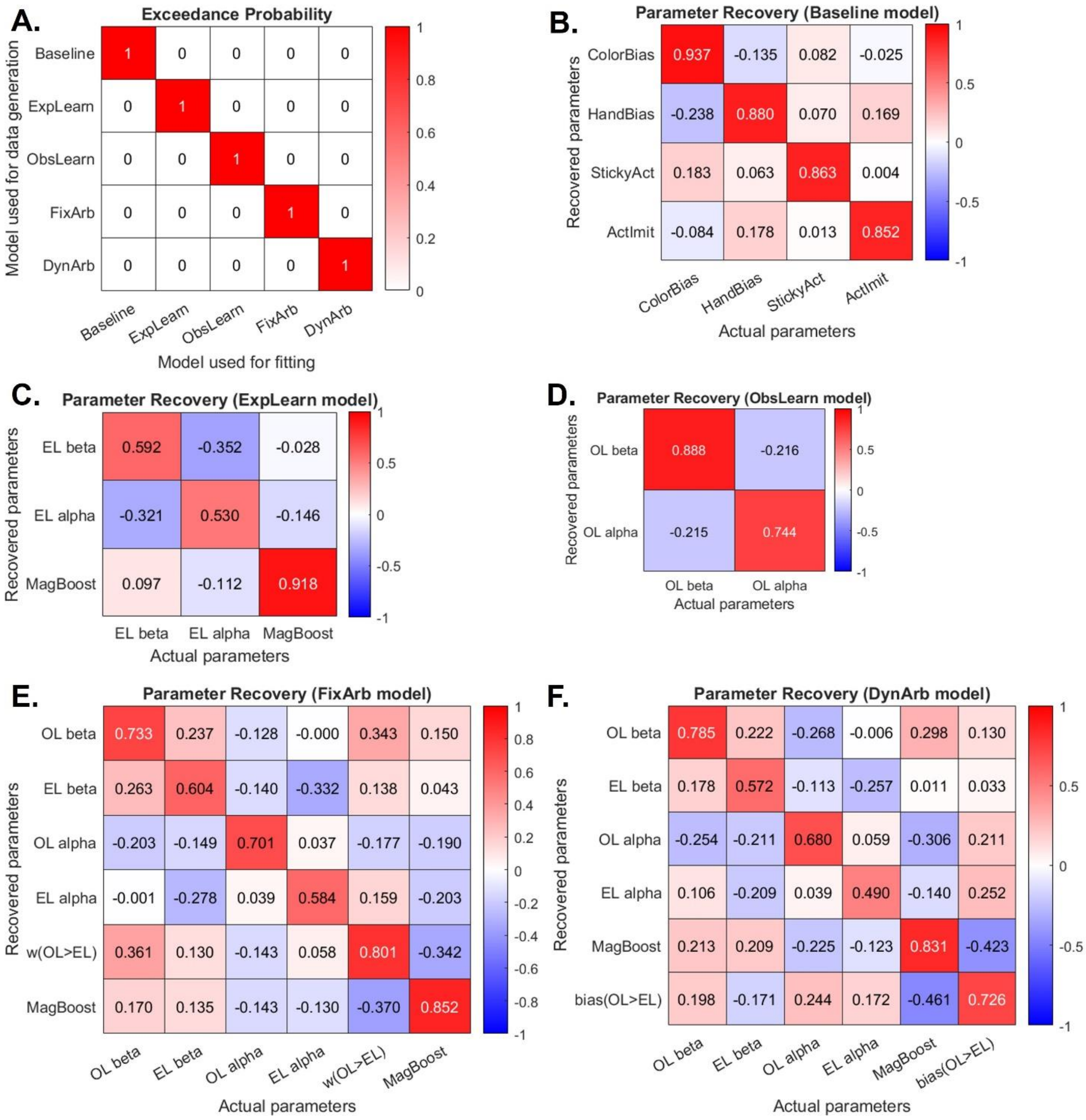

Figure S3. Model and parameter recovery. A. Model recovery was performed by generating data (N=100 simulated datasets) from each model, then performing model fitting and comparison using hierarchical Bayesian inference over the 5 models in the set. Exceedance probabilities are shown, indicating perfect recovery and no confusion between models. **B-F.** Parameter recovery was also performed for each of the models using Study 1 parameter values (N=126). Darker colors in the diagonal of each matrix shows high recovery (R(126)>0.49) for all parameters, higher than any existing cross-parameter correlations (R(126)<0.36).

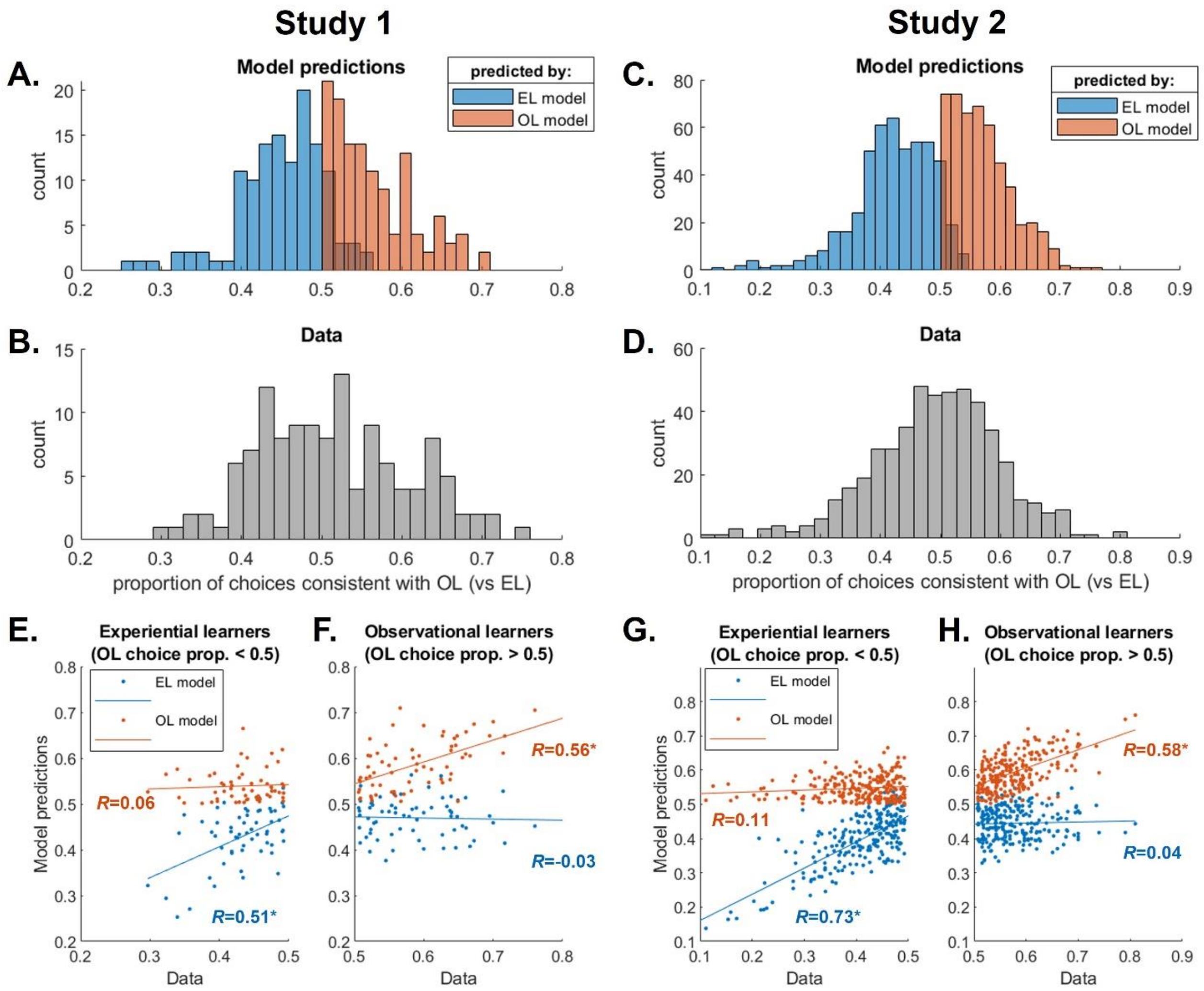

Figure S4. Posterior predictive checks of EL and OL models. A-D. Choice data was generated from each participant's best-fit parameters and from each model, then the proportion of those choices consistent with OL (vs EL) behavior was calculated for each generated choice set and averaged across 1,000 simulations. Those average simulated OL choice propensity values, for Study 1 (**A**) and Study 2 (**C**), are plotted in a histogram across participants, with data generated by the EL model in blue and data generated by the OL model in orange. Plotted for comparison is the histogram of the actual participants' data, for Study 1 (**B**) and Study 2 (**D**). Note that this is the same data as **Fig. 2C-D**, but plotted in histogram format. **E-H.** Cross-participants correlations between OL choice propensity data and model predictions, plotted separately for participants with OL choice propensity data smaller than 0.5 (~Experiential learners), in Study 1 (**E**, N=58) and Study 2 (**G**, N=234), and participants with OL choice propensity greater than 0.5 (~Observational learners), in Study 1 (**F**, N=67) and Study 2 (**H**, N=238). In both studies, behavior of experiential learners was captured by the EL model (Study 1: R(58)=0.51, P<0.001; Study 2: R(234)=0.73, P<0.001), but not by the OL model (Study 1: R(58)=0.06, P=0.67; Study 2: R(234)=0.13, P=0.05), while behavior of observational learners was captured by the OL model (Study 1: R(67)=0.56, P<0.001; Study 2: R(238)=0.58, P<0.001), but not by the EL model (Study 1: R(67)=-0.03, P=0.78; Study 2: R(238)=0.03, P=0.63).

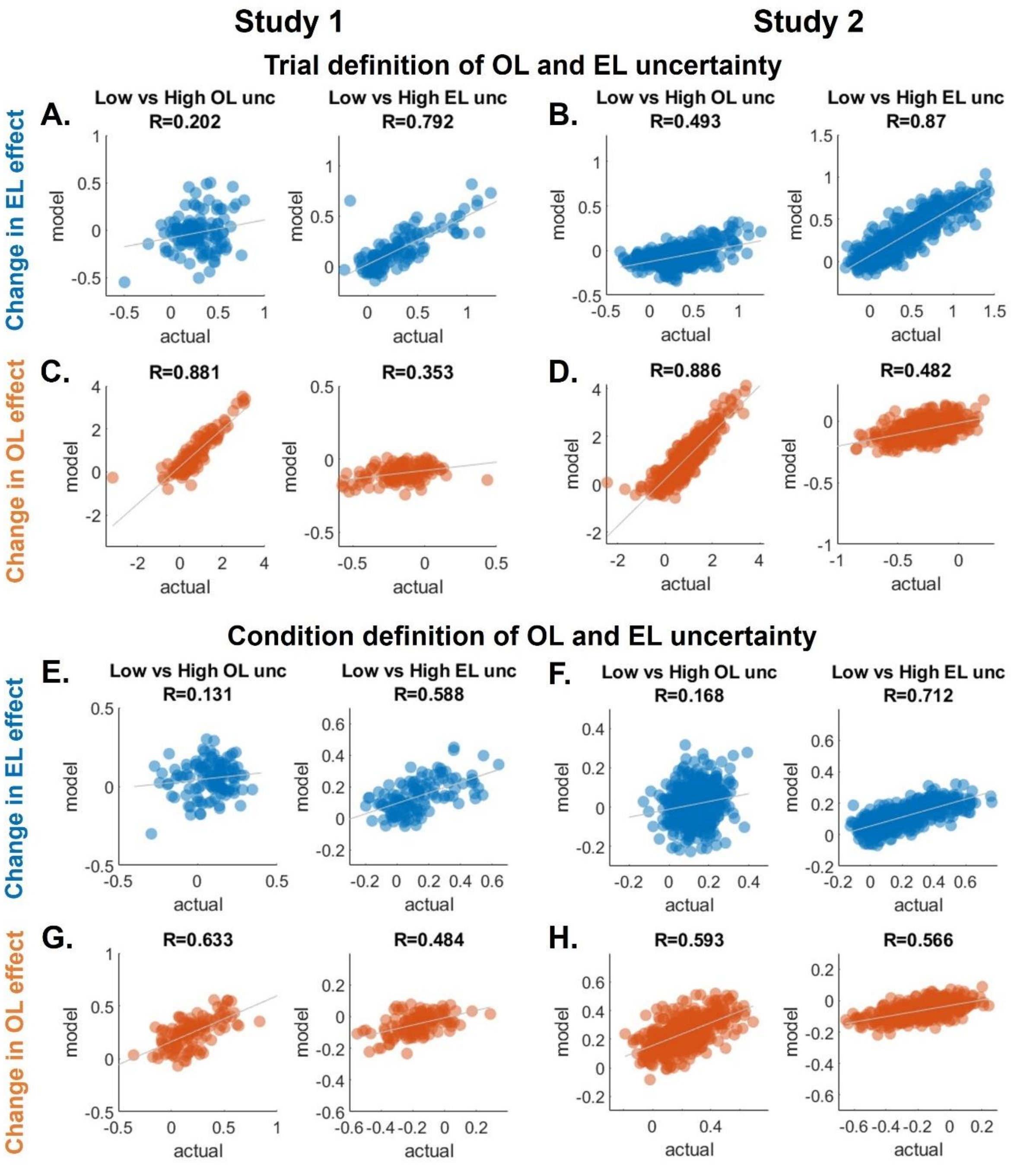

Figure S5. Correlation between actual and model-generated change in ME-GLM effects with uncertainty. The ME-GLMs computing the interactions between each strategy (EL effect, OL effect) and each uncertainty type (EL uncertainty, OL uncertainty) were run on data generated by the dynamic arbitration model as well as on the actual data (see main text **Fig. 4E-H** for effects). We then computed, for each participant, the difference in EL effect (**blue, A-B & E-F**) and the difference in OL effect (**orange, C-D & G-H**) between low and high OL uncertainty (**left panels**) and between low and high EL uncertainty (**right panels**). We repeated this analysis using the *trial* definitions of uncertainty (**A-D**) and the *conditions* defined by the task design (**E-H**). We found that in both cases, the dynamic arbitration model was able to recover the effects from the data, with stronger recovery for the effect of uncertainty on its corresponding strategy (i.e. EL uncertainty on EL effect – **blue, right panels** – and OL uncertainty on OL effect – **orange, left panels**) and stronger recovery when using uncertainty trials compared to uncertainty conditions to define uncertainty. Study 1 (**A, C, E, G**): N=126 independent participants; Study 2 (**B, D, F, H**): N=493 independent participants.

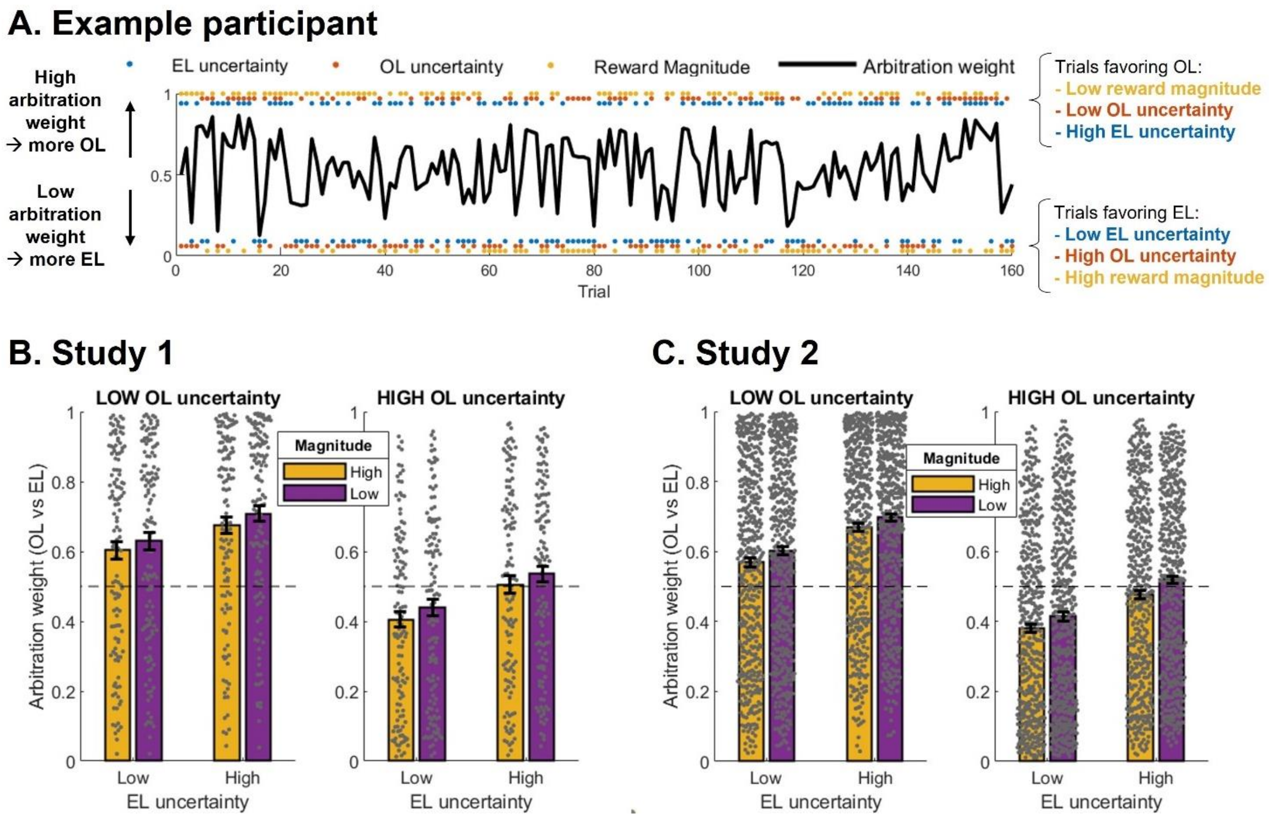

Figure S6. Dynamic variation in arbitration weight and sensitivity to uncertainty and magnitude. Trial-by-trial values of the arbitration weight ($\omega_{OL>EL}(t)$) were extracted from fitting the dynamic arbitration model to each participant's choice behavior. **A.** Those values are plotted across trials for an example participant: high values represent more reliance on OL and low values more reliance on EL. Trial type definitions of EL uncertainty (blue dots), OL uncertainty (orange dots) and reward magnitude (yellow dots) are depicted. **B-C.** To statistically test whether arbitration weight values vary as expected with those changes in uncertainty and magnitude, they were averaged for each of 8 trial types defined by EL uncertainty (low or high), OL uncertainty (low or high) and past reward magnitude (low or high), for Study 1 (**B**, N=126) and Study 2 (**C**, N=493). Each dot is an individual participant; error bars represent SEM. In both studies, there were significant main effects of EL uncertainty (Study 1: $F(1,882)=567$, $P<0.001$, $\eta_p^2=0.391$; Study 2: $F(1,3451)=2825$, $P<0.001$, $\eta_p^2=0.450$), OL uncertainty (Study 1: $F(1,882)=2540$, $P<0.001$, $\eta_p^2=0.742$; Study 2: $F(1,3451)=9899$, $P<0.001$, $\eta_p^2=0.742$) and reward magnitude (Study 1: $F(1,882)=73.3$, $P<0.001$, $\eta_p^2=0.077$; Study 2: $F(1,3451)=333$, $P<0.001$, $\eta_p^2=0.088$) on arbitration weight, thus validating the dynamic arbitration scheme and the definition of high versus low uncertainty/magnitude trials.

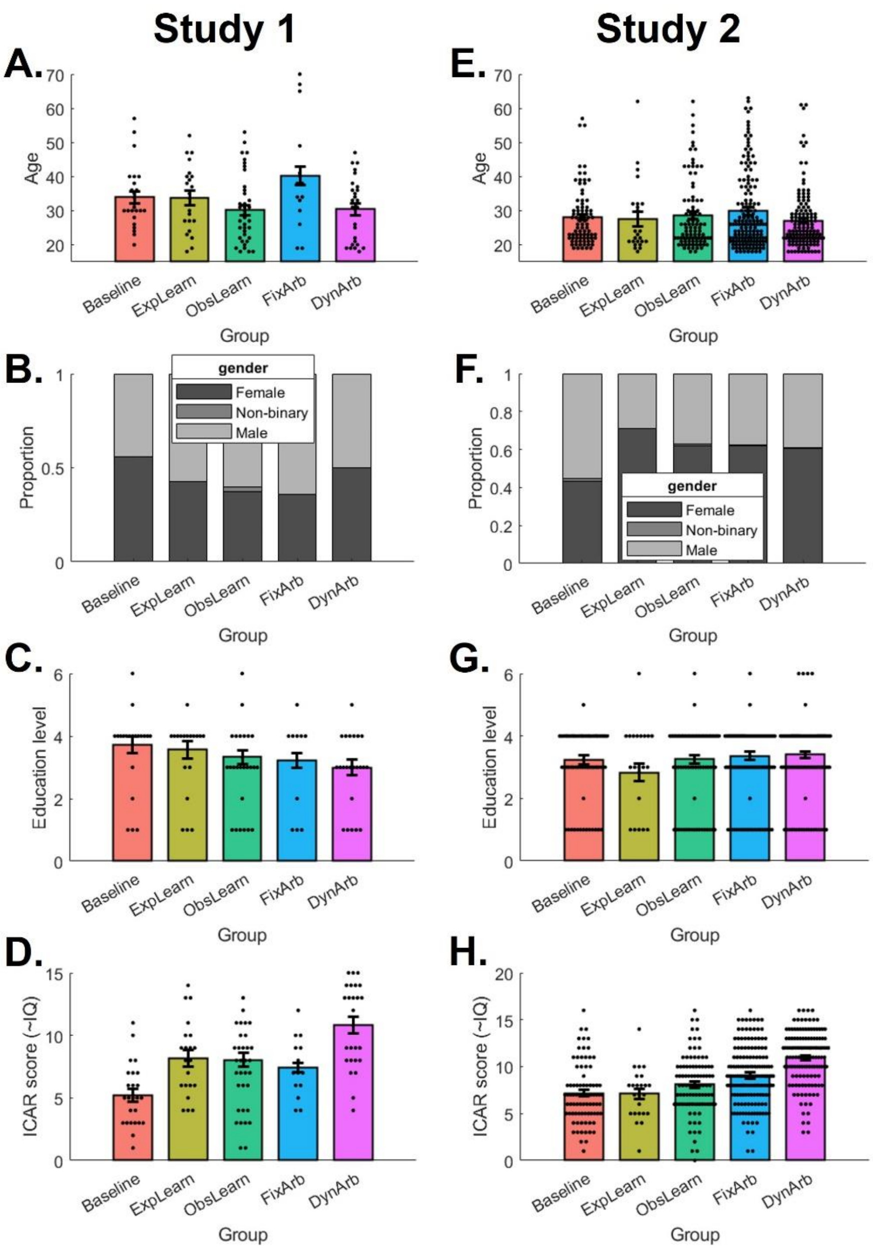

Figure S7. Group differences in age, gender, education, and cognitive ability. Mean and standard error of age (**A, E**), education (**C, G**), and ICAR scores (**D, H**), as well as gender proportions (**B, F**) are plotted for each of the five groups, for Study 1 (**A-D**, $N_{Baseline}$=25, $N_{ExpLearn}$=21, $N_{ObsLearn}$=40, N_{FixArb}=14, N_{DynArb}=26) and Study 2 (**E-H**, $N_{Baseline}$=83, $N_{ExpLearn}$=24, $N_{ObsLearn}$=95, N_{FixArb}=160, N_{DynArb}=131) separately. There was a significant group difference in age in Study 1 (F(4,121)=2.81, P=0.028, η_p^2=0.085, **A**) but not Study 2 (F(4,488)=1.49, P=0.20, η_p^2=0.012, **E**), a significant difference in male vs female distributions in Study 2 (χ^2(4)=10.54, P=0.032, **F**), but not Study 1 (χ^2(4)=2.69, P=0.61, **E**), no statistically significant difference in education level (Study 1: F(4,121)=1.03, P=0.39, η_p^2=0.033, **C**; Study 2: F(4,484)=1.16, P=0.32, η_p^2=0.010, **G**), and a significant difference in ICAR scores in both studies (Study 1: F(4,120)=10.62, P<0.001, η_p^2=0.262, **D**; Study 2: F(4,488)=26.16, P<0.001, η_p^2=0.177, **H**), mainly driven by higher ICAR scores in the dynamic arbitration group.

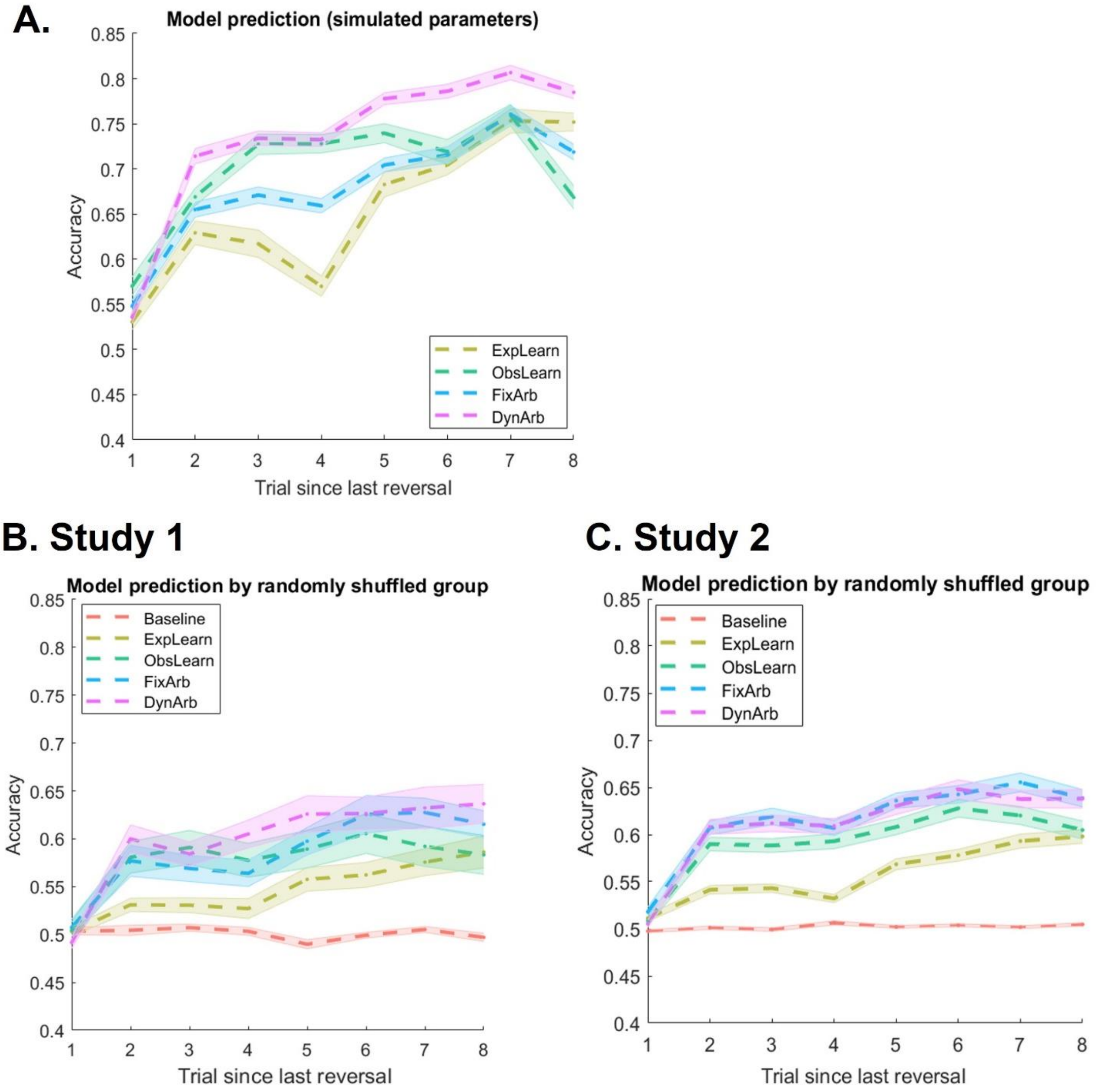

Figure S8. Simulated learning curves. A. To ensure that the four learning models (excluding Baseline) were able to learn the task, independent of the participants' best-fitting parameters, we generated data from those four models using the same simulated parameter values (N=100 independent simulations) across models and plot the resulting learning curves. The curves reflect the choice accuracy on the first 8 trials following each reversal (similar to **Fig. 2A-B**). The shaded area represents standard errors across the 100 simulations. **B-C.** Learning curves were also computed from model-generated data using participants' best-fitting parameters but randomly shuffled group membership (i.e. assigning each participant to a group at random then using this group's corresponding model), in Study 1 (**B**, N=25 or N=26 per group depending on random assignment) and Study 2 (**C**, N=98 or N=99 per group depending on random assignment).

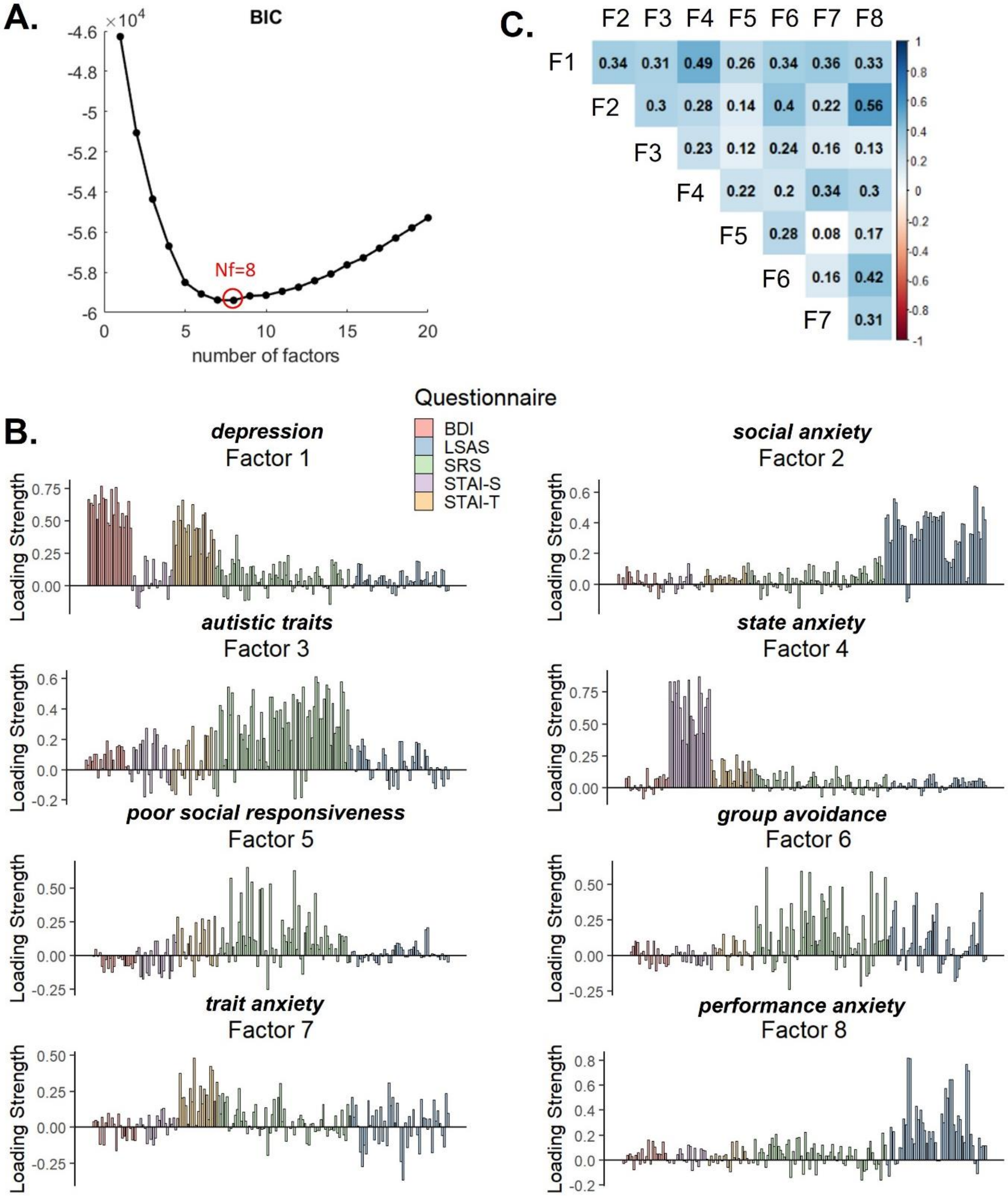

Figure S9. Factor analysis over questionnaire items. A. To determine the optimal number of factors, the factor analysis was performed for the number of factors ranging from 1 to 20. BIC was extracted from each model, indicating that 8 factors provided the best fit (lowest BIC) to the data. Specifically, BIC for 8 factors was -59393, while BIC for 7 and 9 factors (the next two closest values) was -59379 and -59172, respectively. **B.** Factor loadings are shown for each item and each factor, colored by questionnaire. Given the items with highest loading strength, names are provided for each factor to reflect its most representative meaning. **C.** Correlation between factor scores across participants (N=568), with R values provided in each cell of the correlation matrix. The most highly correlated factors were (i) social and performance anxiety (R(568)=0.56, P<0.001), (ii) depression and state anxiety (R(568)=0.46, P<0.001), and (iii) group avoidance and performance anxiety (R(568)=0.42, P<0.001).

Supplementary Discussion

Reward magnitude effects

In addition to the predicted effect of EL and OL uncertainty trials as drivers of arbitration[19], we also found that reward magnitude (but not the variance in magnitude) substantially impacted behavior, by favoring EL over OL when the magnitude of recent rewards was large. This finding, while not predicted from our initial hypotheses, was nevertheless robust and found in both studies. A previous study has reported effects of outcome magnitude on model-based/model-free arbitration[1]. Outcome value effects on arbitration can be predicted by some theories of arbitration such as Expected Value of Control theory[2], among others[3-4], according to which cognitively expensive but more accurate strategies such as model-based RL are more likely to be selected over a cheaper but less accurate model-free counterpart when stakes are high. In the present study, the applicability of such theories is less clear, and if anything, the incentive to utilize an OL strategy on top of an EL strategy would potentially be greater if stakes are higher according to such reward/cost tradeoff approaches. Instead, we found EL was more engaged when stakes are higher. A potential explanation for these magnitude effects is that they are the result of increased attentional salience induced by high rewards which boosts the learning strategy most closely associated with those rewards (here EL). Since our task design was not directly optimized to assess the effect of magnitude, future work will be needed to better understand the computational mechanisms by which reward magnitude impacts arbitration, especially in the context of arbitration across learning domains.

References

1. Kool, W., Gershman, S. J. & Cushman, F. A. Cost-Benefit Arbitration Between Multiple Reinforcement-Learning Systems. Psychol. Sci. 28, 1321–1333 (2017).

2. Shenhav, A., Botvinick, M. M. & Cohen, J. D. The Expected Value of Control : An Integrative Theory of Anterior Cingulate Cortex Function. Neuron 79, 217–240 (2013).

3. Keramati, M., Dezfouli, A. & Piray, P. Speed/Accuracy Trade-Off between the Habitual and the Goal-Directed Processes. PLoS Comput. Biol. 7, e1002055 (2011).

4. Pezzulo, G., Rigoli, F. & Chersi, F. The Mixed Instrumental Controller: using Value of Information to combine habitual choice and mental simulation. Front. Psychol. 4, 92 (2013).
