# Supplementary material for: Heterogeneity in strategy use during arbitration between experiential and observational learning
Source: Nat Commun. 2024 May 24;15:4436. doi: 10.1038/s41467-024-48548-y (PMC11126711; doi:10.1038/s41467-024-48548-y)
Supplement: Supplementary file 3 — Reporting Summary [file 41467_2024_48548_MOESM3_ESM.pdf]

Corresponding author(s): Caroline J. Charpentier

Last updated by author(s): Feb 29, 2024

Reporting Summary

Nature Portfolio wishes to improve the reproducibility of the work that we publish. This form provides structure for consistency and transparency in reporting. For further information on Nature Portfolio policies, see our Editorial Policies and the Editorial Policy Checklist.

Statistics

For all statistical analyses, confirm that the following items are present in the figure legend, table legend, main text, or Methods section.

n/a	Confirmed	
☐	☒	The exact sample size (*n*) for each experimental group/condition, given as a discrete number and unit of measurement
☐	☒	A statement on whether measurements were taken from distinct samples or whether the same sample was measured repeatedly
☐	☒	The statistical test(s) used AND whether they are one- or two-sided<br>*Only common tests should be described solely by name; describe more complex techniques in the Methods section.*
☐	☒	A description of all covariates tested
☐	☒	A description of any assumptions or corrections, such as tests of normality and adjustment for multiple comparisons
☐	☒	A full description of the statistical parameters including central tendency (e.g. means) or other basic estimates (e.g. regression coefficient) AND variation (e.g. standard deviation) or associated estimates of uncertainty (e.g. confidence intervals)
☐	☒	For null hypothesis testing, the test statistic (e.g. *F*, *t*, *r*) with confidence intervals, effect sizes, degrees of freedom and *P* value noted<br>*Give P values as exact values whenever suitable.*
☐	☒	For Bayesian analysis, information on the choice of priors and Markov chain Monte Carlo settings
☐	☒	For hierarchical and complex designs, identification of the appropriate level for tests and full reporting of outcomes
☐	☒	Estimates of effect sizes (e.g. Cohen's *d*, Pearson's *r*), indicating how they were calculated

Our web collection on statistics for biologists contains articles on many of the points above.

Software and code

Policy information about availability of computer code

Data collection	Data collection was performed online, the task and questionnaires were coded up into a HTML URL, using custom Javascript code and plugins from jspsych versions 6.0.5. and 6.1.0. A demonstration of the task is available here: https://obsexplearn.web.app/ and the code for running the experiment is available on Github: https://github.com/ccharpen/OL_EL_behavior
Data analysis	Data was analyzed using Matlab (R2020b) and R (version 4.1.2). All code for data analyses and figure/table generation is available at: https://github.com/ccharpen/OL_EL_behavior and under the following DOI: https://doi.org/10.5281/zenodo.10695037.

For manuscripts utilizing custom algorithms or software that are central to the research but not yet described in published literature, software must be made available to editors and reviewers. We strongly encourage code deposition in a community repository (e.g. GitHub). See the Nature Portfolio guidelines for submitting code & software for further information.

Data

Policy information about availability of data

All manuscripts must include a data availability statement. This statement should provide the following information, where applicable:

- Accession codes, unique identifiers, or web links for publicly available datasets
- A description of any restrictions on data availability
- For clinical datasets or third party data, please ensure that the statement adheres to our policy

The raw (trial-by-trial) and summary (participant-level) data generated in this study are available at: https://github.com/ccharpen/OL_EL_behavior

Research involving human participants, their data, or biological material

Policy information about studies with human participants or human data. See also policy information about sex, gender (identity/presentation), and sexual orientation and race, ethnicity and racism.

Reporting on sex and gender	We report gender data in the manuscript. During the demographic questions at the beginning of the study, participants were asked to provide their gender by selecting one of the following options: "Female", " Male", "Non-specific".<br>Sex data is available from Prolific screeners in some of the participants, but our analyses focus on gender as a variable taken into account in the analyses. Neither sex nor gender were considered key variables of interest in this study - yet we controlled for gender in statistical analyses to ensure our results were not driven by gender. Sample size broken down by gender is reported below in the "Behavioural & social sciences study design" section of this reporting summary.
Reporting on race, ethnicity, or other socially relevant groupings	Race, ethnicity and highest completed level of education were collected through demographic questions at the beginning of the study.<br>For race, participants were asked to select all the options that apply among the following: "American Indian or Alaska Native", "Asian", "Black or African-American", "Native Hawaiian or Other Pacific Islander", "White", "More than one race", "Unknown or not reported".<br>For ethnicity, participants were asked to select one option among: "Hispanic or Latino", "Not Hispanic or Latino", "Unknown or not reported".<br>For level of education, participants selected one of the following: "Did not complete high school", "High school diploma/ GED", "Currently in college/university", "Associate's degree (2 yr)", "Bachelor's degree (4 yr)", "Master's degree", "Doctoral degree". Answers in the order described above were recoded from 0 ("Did not complete high school") to 6 ("Doctoral degree") in order to be used as a continuous variable in the analyses.
Population characteristics	See "Behavioural & social sciences study design" section below.
Recruitment	Participants were recruited online from Prolific Academic (https://www.prolific.co), and sampling occured randomly out of eligible participants as a way to mitigate selection biases. Yet we cannot rule out self-selection biases such as biases in the overall population who sign up to participate in online studies on Prolific (e.g., more females than males overall), or possible biases induced by the subset of participants who drop out of the experiment (e.g., participants from lower socio-economic backgrounds may experience less reliable internet connections and drop out of studies at a higher rate).
Ethics oversight	All participants provided informed consent. The study was deemed exempt from full IRB review after an initial review by the Caltech Institutional Review Board due to being judged to be of minimal risk to participants and meeting several other criteria required for an exempt status.

Note that full information on the approval of the study protocol must also be provided in the manuscript.

Field-specific reporting

Please select the one below that is the best fit for your research. If you are not sure, read the appropriate sections before making your selection.

☐ Life sciences ☒ Behavioural & social sciences ☐ Ecological, evolutionary & environmental sciences

For a reference copy of the document with all sections, see nature.com/documents/nr-reporting-summary-flat.pdf

Behavioural & social sciences study design

All studies must disclose on these points even when the disclosure is negative.

Study description	This is an online behavioral study including exclusively quantitative data (choice responses and reaction time during a task, as well as likert-scale self-report questionnaires).
Research sample	In total, we tested 621 participants across 2 study samples (346 female, 270 male, 5 non-binary, mean age = 29.38 +/-10.1 years). Participants were recruited on an online data collection platform, Prolific Academic (https://www.prolific.co), and reported US as their country of residence, fluency in English, age between 18 and 65, no literacy difficulty, and normal or corrected-to-normal vision. Sampling was random across eligible Prolific participants, and therefore is not representative. The sample was chosen to be from the general population, able to understand and perform the task (English understanding, vision, etc) and spanning a wide age range.
Sampling strategy	The first study included 128 participants, a sample size in line with existing online study samples. The second study included 493 participants in order to ensure sufficient statistical power to replicate Study 1's findings and large enough to be able to characterize individual differences in relation to psychiatric symptom dimensions, where we expect effect sizes to be small (99% power to detect a correlation of r=0.2 or greater).
Data collection	The study took place online - participants on Prolific were required to take the study from a desktop computer or laptop (no smartphone or tablet). They were presented with stimuli on the screen and had to press one of two keyboard keys to answers. At the end of the study they completed self-report questionnaires by clicking on their desired option for each question. Participants' responses were saved on a dedicated secure database on Google Firebase.<br>Participants are assumed to be alone with their computer (although we cannot control their environment) and do not directly

	interact with the experimenter. There were no between-subjects experimental conditions, and the researcher was not blinded to the study hypothesis.
Timing	Study 1 data was collected between December 2-8, 2020. Study 2 data was collected between July 26th and September 8th, 2021.
Data exclusions	Task-based exclusions: Two participants were excluded from Study 1 for missing more than 25% of trials. No participant in Study 2 met this exclusion criterion.<br>Questionnaire-based exclusions: 51 participants were excluded across the pooled sample for various pattern of careless responding on the questionnaires (low (Z<-2) intra-individual response variability, split-half reliability or psychometric synonym, or high (Z>2) psychometric antonym). Those criteria were established using R's careless package (Rouder et al, 2009) and the same as a recent published study (Zbozinek et al, 2021).
Non-participation	In total, 87 participants dropped out or declined participation. The reason for those is unknown, as participants can withdraw from the study at any time - possible explanations include lack of time to finish the study or loss of internet connection.
Randomization	Each study was independent and included repeated measures only; therefore, there was no allocation to experimental groups.

Reporting for specific materials, systems and methods

We require information from authors about some types of materials, experimental systems and methods used in many studies. Here, indicate whether each material, system or method listed is relevant to your study. If you are not sure if a list item applies to your research, read the appropriate section before selecting a response.

Materials & experimental systems

n/a	Involved in the study
☒	☐ Antibodies
☒	☐ Eukaryotic cell lines
☒	☐ Palaeontology and archaeology
☒	☐ Animals and other organisms
☒	☐ Clinical data
☒	☐ Dual use research of concern
☒	☐ Plants

Methods

n/a	Involved in the study
☒	☐ ChIP-seq
☒	☐ Flow cytometry
☒	☐ MRI-based neuroimaging

Plants

Seed stocks	N/A
Novel plant genotypes	N/A
Authentication	N/A
